# The efficacy and safety of second salvage autologous transplantation in myeloma patients

**DOI:** 10.3389/pore.2024.1611851

**Published:** 2024-07-16

**Authors:** Reka Rahel Bicsko, Renata Nyilas, Robert Szasz, Laszlo Varoczy, Attila Kiss, Miklos Udvardy, Arpad Illes, Lajos Gergely

**Affiliations:** Department of Hematology, Faculty of Medicine, Doctoral School of Clinical Sciences, Institute of Internal Medicine, University of Debrecen, Debrecen, Hungary

**Keywords:** multiple myeloma, salvage therapy, salvage transplantation, second autologous stem cell transplantation, survival

## Abstract

Despite the availability of many novel therapies for multiple myeloma, it remains an incurable disease with relapse fated in almost all patients. In the era of modern agents, second autologous stem cell transplantation still holds its role in patients relapsing after first-line autologous transplant. The authors reviewed a single-center experience with a second auto-SCT for relapsed multiple myeloma. Thirty patients had received a salvage auto-SCT at the institution. The median follow-up after diagnosis was 86 months, and the median time between transplants was 59.1 months. Response before second ASCT was the following: CR – 11 cases, VGPR – 9 cases, PR – 10 cases. Most patients received reduced dose (140 mg/m2) of melphalan as a conditioning regimen for the second auto-SCT. Treatment-related mortality was 3%. With a median follow-up time of 34 months after the second transplant, median progression-free survival was 24 months. The median PFS in the patients achieving CR or VGPR at day 100 after the second transplantation was 32 months. By 15 months, all patients achieved only partial remission progressed, with a median PFS of 8.5 months. During the follow-up period, no MDS or AML developed, and the frequency of second malignancy was also low, 3%. In conclusion, second autologous stem cell transplantation is a well-tolerated and effective treatment option for relapsed multiple myeloma in selected patients, though with a shorter PFS than in first remission.

## Introduction

The survival of myeloma patients has drastically improved during the last two decades, with the introduction of new drugs like proteasome inhibitors and imids. The first improvement in survival was demonstrated by high-dose melphalan therapy with autologous transplantation after first-line induction. In the early 1990s, it was documented that adding autologous transplantation to the front-line chemotherapy increases the progression-free survival (PFS) and overall survival (OS) of myeloma patients, and an 80% estimated 3-year OS could be achieved [1]. Since then, for more than three decades, the role of autologous transplantation still holds its place after first-line induction therapy despite the introduction of newer drugs into induction therapy. It was demonstrated that the deeper remission the patient achieves before the transplantation, the better the PFS after the transplantation. The mortality rate from this procedure has drastically decreased from 10% to less than 1%. In the early 2000s, it was shown that second autologous transplantation may increase the 10-year OS from 20.4% to 35.2%, highlighting the importance of this therapy [2]. Upfront tandem transplantation could achieve a 55% chance of 5-year OS, and the essential survival predictor was the response quality after either transplantation [3]. These results showed that chemotherapy with melphalan adds a survival benefit for myeloma patients. The concept of second autologous transplantation as a consolidation of salvage modality for relapsed myeloma patients was established in that era, with a mortality of 3%. The median event-free survival (EFS) after the first transplant was 15.7 months and 12.9 months after the second transplantation [4].

The long-term results of the Total Therapy III trial have recently been published. The data demonstrates that adding melphalan to myeloma therapy increases survival of patients. The median PFS was 5.6 years, and the 15-year PFS was still 27.9% with an OS of 35.9% [5].

The treatment of relapsed/refractory myeloma has drastically changed during the last 10 years. New proteasome inhibitors, immunomodulators, monoclonal antibodies, bispecific antibodies, and CAR-T cells became available, giving clinicians several choices to treat the patients. All these drugs achieve better PFS for patients, but the price of these therapies is markedly increasing. Also, by utilizing these drugs, we are facing new problems, like the so-called “penta-refractory” myeloma patients, where most of the novel drugs failed. The use of second autologous transplantation to consolidate a remission is still a viable option in selected cases, however there is a big debate whether this modalitiy should still be used. There are several results of using high-dose melphalan with second autologous transplantation. In 2006, the MD. Anderson Cancer Center reported a retrospective analysis with a median PFS of 6.9 months and a median OS of 29 months in a relatively young patient population, with a median age of 52 years [6]. In another report, the median PFS was 8.5 months, and the median OS was 20.7 months after the second autologous transplantation, with a median of 37 months after the first transplantation [7]. Another retrospective study showed that patients who achieve at least 24 months of PFS after the first transplantation do better with a second autograft. The median PFS was 19 months, but patients who relapsed within 24 months after the first transplantation had a median PFS of 9.83 months compared to 17.3 months for later relapsing patients. This was also reflected in the OS as early relapsing patients had a median OS of 28.47 months compared to 71.3 months in the other group [8]. This publication highlighted the importance of selecting patients based on their response to first high-dose melphalan therapy. Another retrospective multivariate analysis also confirmed that the time to progression after the first transplantation and the quality of response before the second transplantation impact the outcome of the salvage transplantation. Patients with at least a PR had a 2-year OS of 85.9% compared to the other patients, whose 2-year OS was 51.3% [9]. In a retrospective study by the Karolinska Institute, the authors compared the results of a second autograft with the results of novel drugs in relapsed patients. They confirmed the superiority of the second autograft as the median OS was 4 years, compared to 3.3 years in the novel drug group [10]. It has also been reported that the timing of a second autologous transplantation is optimal after the first relapse compared to later, as both PFS and OS were significantly better in the first group [11].

These data highlight that autologous transplantation may still hold its place in myeloma therapy and that second transplantation as a salvage option is still a possible option for selected patients.

Second transplantation as a salvage option is not widely used; there are only a few reports on this approach. The authors report their findings on myeloma patients who underwent a second ASCT to provide data on the validity of second salvage autologous transplantation in the era of novel therapies. Authors demonstrate that the survival results are still comparable to what is achievable with novel therapies, with minimal toxicity.

## Study design

This is a retrospective single-center cohort analysis of multiple myeloma patients undergoing second autologous transplantation at the University of Debrecen. All patients were included who underwent a second autologous transplantation with the diagnosis of multiple myeloma between 1 January 2008 and 31 December 2023. The patient’s clinical data was retrospectively collected from the hospital’s medical records. The data was also cross-checked with the data reported towards the European Bone Marrow Transplantation Society (EBMT). All patients agreed on the data collection in a consent form at the time of both transplantations. A second transplant was considered if the patient had received a prior autologous stem cell transplantation, after which they required subsequent treatment due to disease progression. A relapse was defined as a reappearance of serum or urine M-protein by immunofixation or electrophoresis, development of ≥5% plasma cells in the bone marrow or appearance of any other sign of progression based on IMWG criteria [12]. No planned tandem transplantation case was included. Response to transplantation was assigned as the change of burden of myeloma plasma cells in the bone marrow and the amount of circulating monoclonal protein in the sera. May-Grünwald-Giemsa-stained smears and flow cytometric assay was performed to examine the number of plasma cells in the bone marrow. Day 100 after the transplant was used as a landmark point to analyze the response to auto-SCT. Complete response was defined in case of negative immunofixation in the serum and urine, and disappearance of any soft tissue plasmacytomas and 5% or fewer plasma cells in bone marrow aspiration. A very good partial response was stated when at least a 90% reduction of serum M-protein plus urine M-protein level <100 mg per 24 h was confirmed. Partial response was described as ≥50% reduction of serum monoclonal protein level plus reduction in 24 h urinary M-protein >90% or to <200 mg per 24 h. The upper age limit for a second autologous transplant was 75 years with adequate physiological function, according to Karnofsky status scale and Eastern Cooperative Oncology Group performance status, and a life expectancy longer than 6 months.

Heart failure, reduced pulmonary function, chronic respiratory diseases and liver failure were exclusion factors. Renal failure due to cast nephropathy, even hemodialysis dependency, was not an exclusion criterion. The conditioning regimen was full-dose (200 mg/m^2^) or reduced dose (140 mg/m^2^) of melphalan. As per local guidelines, most patients were given a reduced dose of melphalan (140 mg/m^2^) for the second transplantation due to age and previous exposure to this drug. A single intravenous dose of conditioning was given on day −2, followed by peripheral stem cell administration 48 h later. All patients received antiviral prophylaxis with acyclovir, Pneumocystis jirovecii prophylaxis during the peritransplant period with trimethoprim/sulfamethoxazole and antifungal prophylaxis with fluconazole. Filtered, irradiated red blood cells and platelet suspensions were givenif needed. Survival times were calculated on the one hand from the time of diagnosis and on the other hand from the time of the second transplant. OS was defined as the time from diagnosis and time from the second ASCT to death from any cause and PFS was defined as the time from auto-SCT to relapse or progressive disease or death from any cause, whichever came first. PFS from the second transplant was also calculated. Transplant-related mortality was defined as mortality from any cause other than disease progression within 100 days from the date of transplantation.

Statistical analysis was performed using GraphPad Prism 10.1 software. The survival times were calculated using the Kaplan-Meier method. The difference between survival times was compared using the Log-Rank test. Statistical significance was stated if the *p*-value was less than 0.05. The data collection protocol has been approved by the authors’ respective Institutional Review Board for human subjects (IRB reference number: 6739/2024). This study was managed according to the Declaration of Helsinki.

## Results

**FIGURE 1:**
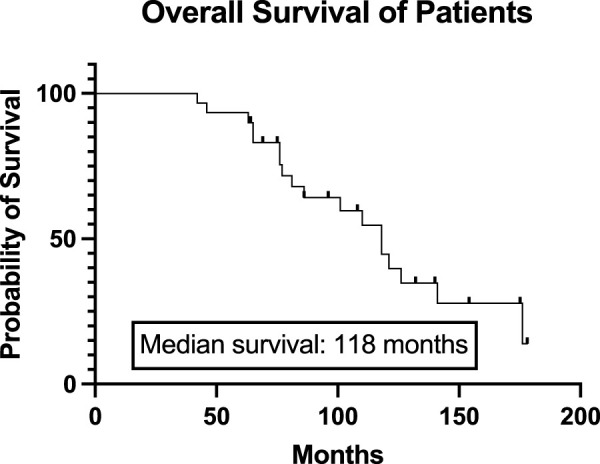
Overall survival of the total study population from the diagnosis. The Kaplan-Meier curve represents the overall survival of all patients from the diagnosis. The median overall survival was 118 months.

Thirty multiple myeloma patients underwent two autologous hematopoietic stem cell transplantations (auto-SCT) in the defined time interval in a single institution. Clinical characteristics at diagnosis and at the time of initial auto-SCT are summarized in Table 1. The median age at diagnosis was 53.5 years (range 37–68). The proportion of men and women was the same (15:15, 50%–50%). M protein was IgG in most cases (50%), IgA in 23.3%, and light chain only disease occurred in 13.3%. The cytogenetic profile of some patients is not available as at the time of these treatments the test was not routinely used. All patients received bortezomib-based induction therapy and a median of 1 (range 1–4) line of treatment before the first transplant. The median time interval between diagnosis and first transplant was 49 months (range 5–129). Almost all patients (29, 97%) received full-dose (200 mg/m^2^) intravenous melphalan conditioning. Complete remission was developed in two-thirds (66.6%) of patients after the initial auto-SCT. Median follow-up after diagnosis was 86 months, while median overall survival was 118 months in the defined population (Figure 1). Median progression-free survival was 39 months after the first autologous transplant.

**TABLE 1:** Patients’ characteristics at the diagnosis and first autologous stem cell transplantation.

*Variable*	*Value*
Gender	15 male, 15 female
Age at diagnosis (years)	53.5 years (37–68)
Multiple myeloma disease types	
IgG IgA kappa light chain lambda light chain	15 (50%) 7 (23.3%) 4 (13.3%) 4 (13.3%)
ISS stage at diagnosis	
No data Stage I Stage II Stage III	5 9 10 6
Interval between diagnosis and first auto-SCT	49 months (5–129)
Status of disease before first auto-SCT	
complete response very good partial response partial response	11 (37%) 12 (40%) 7 (23%)
Conditioning regimen	
Full dose melphalan (200mg/m2) Reduced dose melphalan (140mg/m2)	29 (97%) 1 (3%)
Response 100 days after first auto-SCT	
complete response very good partial response partial response	20 (66.7%) 8 (26.7%) 2 (6.6%)

The table lists patients’ data at the time of diagnosis and at the time of first stem cell transplantation. SCT, stem cell transplantation.

Patients’ clinical characteristics at the time of the second transplantation are shown in Table 2. The median follow-up after the second transplantation was 34 months (range 11–110). The selection criteria for the second autologous transplantation was no progression within 24 months after the first transplant and inability to continue bortezomib due to toxicity. In one case, the second autologous transplant was performed within 24 months of the first transplant, in the best-achieved response status, because innovative drugs were not available at the time. Median age at second transplantation was 59 years (range 43–73). The median time from diagnosis to second auto-SCT was 94 months (range 27–305), and the median interval between the two transplantations was 59.1 months (range 19–138). Patients received a median of 2 (range 2–6) lines of salvage therapy after the first transplantation. The most commonly used salvage regimens were VRD (bortezomib, lenalidomide, dexamethasone) in 11 cases, CRD (carfilzomib, lenalidomide, dexamethasone) in 7 cases, VTD-PACE (bortezomib, thalidomide, dexamethasone, cisplatin, doxorubicin, cyclophosphamide, etoposide) in 6 cases, Vel-Dex (bortezomib-dexamethasone) in 6 cases, and daratumumab-based therapy in 4 cases (2 DRD–daratumumab, lenalidomide, dexamethasone, 2 DPD–daratumumab, pomalidomide, dexamethasone). There was no difference in PFS between patients who received 1 line of therapy or more than 1 line of therapy before the second transplantation (median PFS 32 months vs. 24 months *p* = 0.469). All second transplantations were performed using peripherally collected stem cells. In 17 cases, the stored frozen stem cells from the collection before the first transplant were used, and the other 13 patients were re-mobilized with G-CSF, and stem cells were collected and were frozen. There was no difference in progression-free survival between the two groups. The median engraftment was 9 days in the stored PBSC group compared to 10 days in the second mobilization and collection group, but the results were not statistically significant (data not shown). 11 patients entered the transplant in CR, 9 patients in VGPR, while 10 patients achieved only a partial response.

**TABLE 2:** Patients’ characteristics at the time of second autologous stem cell transplantation.

*Variable*	*Value*
Age at salvage transplantation	59 years (43–73)
Interval between diagnosis and salvage auto-SCT	94 months (27–305)
Lines of therapy before salvage auto-SCT	
2 3 4 5 6	17 (56.7%) 4 (13.3%) 4 (13.3%) 3 (10%) 2 (6.7%)
Status of disease before salvage auto-SCT	
complete response very good partial response partial response	11 (36.7%) 9 (30%) 10 (33.3%)
Conditioning regimen	
Full dose melphalan 200 mg/m2 Reduced dose melphalan (140 mg/m2)	8 (26.7%) 22 (73.3%)
Treatment related mortality	1 (3%)
Response 100 days after salvage auto-SCT	
complete response very good partial response partial response	18 (60%) 8 (26.7%) 4 (13.3%)
Maintenance therapy	n = 14
lenalidomide	11
ixazomib	2
lenalidomide + bortezomib + dexamethasone	1

The table lists the patients’ detailed data at the time of the second autologous stem cell transplantation. SCT, stem cell transplantation.

**FIGURE 2:**
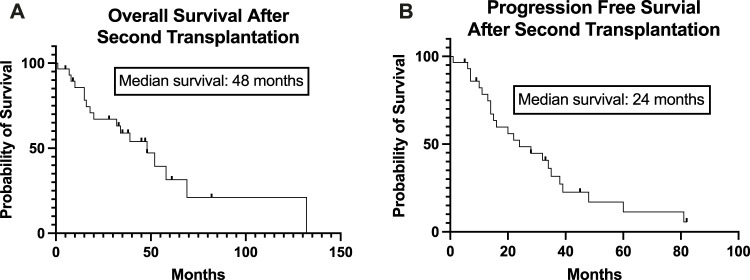
Overall and Progression-Free Survival of the patients from the time of the second stem cell transplantation. The Kaplan-Meier estimated overall survival **(A)** and progression-free survival **(B)** of the study population from the time of the second stem cell transplantation is presented. The median overall survival was 48 months, and the median progression-free survival was 24 months.

**FIGURE 3:**
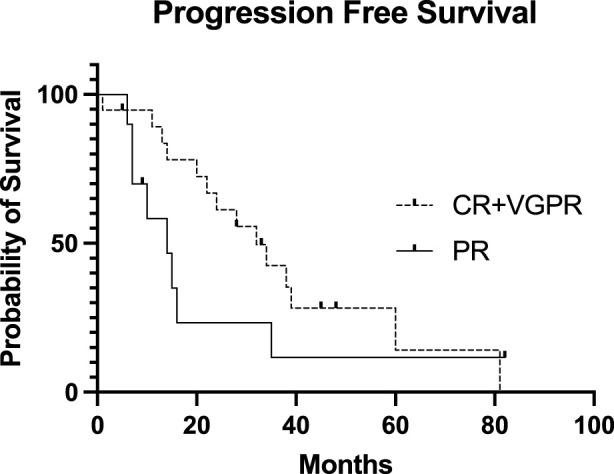
Progression-free Survival of the patients after the second stem cell transplantation based on pretransplant disease status. The Kaplan-Meier estimated progression-free survival (PFS) presented after the second stem cell transplantation based on the pretransplant disease status. The median PFS in the CR + VGPR pretransplant group was 32 months, while the median PFS in the PR pretransplant group was 14 months (*p* = 0.182). CR, complete remission; VGPR, very good partial remission; PR, partial remission.

**FIGURE 4:**
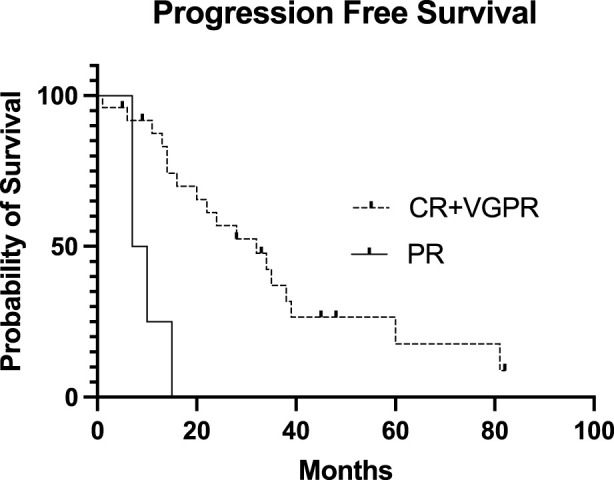
Progression-free Survival of the patients after the second stem cell transplantation based on the day 100 post-transplant disease status. The Kaplan-Meier estimated Progression-Free Survival (PFS) presented after the second stem cell transplantation based on the day 100 post-transplant disease status. The median PFS in the CR + VGPR post-transplant group was 32 months, and the median PFS in the PR post-transplant group was only 8.5 months (*p* = 0.0006). CR, complete remission; VGPR, very good partial remission; PR, partial remission.

The conditioning regimen was full-dose melphalan (200 mg/m^2^) in 8 patients, while a reduced dose (140 mg/m^2^) was used in 22 cases. The two different conditioning regimens did not statistically influence the PFS (*p* = 0.4732, data not shown). The median time to neutrophil engraftment after stem cell infusion was 9 (8–13) days. PFS was not statistically significant based on the engraftment days (cut-off: 9 days, *p* = 0.822, data not shown). Transplant-related mortality was 3%, as one patient died during the first 100 days due to nosocomial pneumonia at day 32. 60% of the patients (18) achieved complete remission evaluated 100 days after salvage transplantation. Of the 10 patients in PR entering the second transplantation, 3 were converted to VGPR and 3 to CR. Only 4 patients remained in PR. Fourteen (46.7%) patients received maintenance therapy after the second transplantation, of which the most common was lenalidomide monotherapy. There was no significant difference between PFS (*p* = 0.836) and OS (*p* = 0.773) based on the use of any maintenance therapy or not (data not shown). Median progression-free survival after the second auto-SCT (PFS-ASCT2) was 24 months, while median overall survival (OS-ASCT2) was 48 months (Figure 2). The median PFS in patients entering the second transplant in CR or VGPR was 32 months, compared to 14 months in the PR patients (*p* = 0.182, NS). The median PFS (PFS-ASCT2) in the patients achieving CR or VGPR at day 100 after the second transplantation was significantly higher at 32 months, compared to 8.5 months in the PR group (*p* = 0.0006) (Figures 3, 4). During the follow-up period, a second malignancy developed in the form of lung cancer in one case. There were no cases of myelodysplastic syndrome (MDS) or acute myeloid leukemia (AML) during the follow-up.

## Discussion

The authors conducted a single-center retrospective review of all multiple myeloma patients who received a second autologous stem cell transplantation between 2008 and 2023 to report the efficacy and safety of this therapy. In the analysis, second autologous stem cell transplantation appears to be a well-tolerated and effective treatment option for relapsed multiple myeloma, even in a heavily pretreated cohort. Most patients (22, 73%) received a reduced dose of melphalan at 140 mg/m^2^ for the second ASCT. There was no significant difference in PFS or OS between patients who received reduced or full-dose melphalan. This suggests that salvage autologous stem cell transplant may still be effective in advanced age, renal insufficiency, or other comorbidities. Merely one patient suffered a treatment-related mortality.In agreement with the literature, the authors state that CR or VGPR is the optimal pre-transplant condition for better survival [13, 14], but patients entering the transplant in PR may also benefit from the transplant. In the reported case series, of the 10 patients proceeding to the second auto-SCT in PR, 6 (60%) converted to VGPR or CR. Based on the author’s limited case series, the post-transplant PR is not an acceptable result. These patients require additional active anti-myeloma therapy to improve survival.

As reported in other analyses, the response to the second transplant appears to be shorter than the first autologous-SCT, with a median PFS of 39 vs. 24 months in our study. The median PFS achieved after the second transplantation is slightly better than reported in other cohorts, in which the median PFS was 20.4 months [15, 16]. Another large CIBMTR study analyzed the role of maintenance therapy after the second auto-SCT in myeloma in 522 patients treated in 92 centers [17]. They reported that maintenance significantly improved PFS (27.8%) and OS (9.8%) at 5 years, compared to no maintenance. This was not reflected in this cohort; the PFS at 5 years was 17% for the whole group, and no difference was found between the subgroups, possibly due to a much smaller sample size. The frequency of second malignancy was lower in this cohort (3%) compared to the reported incidence of 5%–7%, possibly because of the small sample size and shorter follow-up period.

The use and timing of second autologous stem cell transplantation for multiple myeloma have been published by several authors with different outcomes. In the early 2000s, Quazilbash et al. reported that both auto-and allografting is feasible in case of relapsing after the first transplant. Olin et al. concluded that patients who received more than five lines of prior treatment are unlikely to benefit significantly from a second transplant [7]. Fermand et al. did not provide evidence of second auto-SCT’s superiority over conventional chemotherapy [18]. Another Italian cohort demonstrated that two autografts are superior to one auto-transplant [19]. One Tunisian study showed that a single auto-SCT followed by 6 months of thalidomide maintenance therapy was significantly superior to two autologous stem cell transplants without maintenance therapy in OS and EFS [20]. For these reasons, the reported data validates that the second autologous transplantation may be a rational therapeutic option for selected myeloma patients. The results presented are comparable to salvage treatment with bispecific antibodies. The use of CAR-T cells in refractory myeloma may result better survival, but a French real-world analysis of ide-cel in refractory myeloma resulted in a median PFS of 12.5 months and median OS of 20.8 months, which is comparable to our results [21].

Several limitations must be considered in the interpretation of the results. First, the retrospective design is subject to inherent selection bias of non-randomized retrospective data. Second, the study needs to provide evidence on the timing of the second auto-transplant. Also, the patients selected for a second auto-SCT did have a minimum of 24 months response after the first transplantation, which is an obvious selection bias of patients. Bigger sample size and complete data on known prognostic factors and cytogenetics were needed, as FISH testing was not commonly available for the earlier cohort years. Furthermore, long study periods with different eras of diagnostic criteria, as well as therapy with potentially different management approaches, are involved. The results of this study are also limited by the relatively small cohort of heterogeneous patients and the heterogeneity of the prior treatments.

However, the reported study’s real-life results are significant in the novel agent era and the context of CAR-T therapy, as the decision-making for heavily pretreated patients or patients with advanced disease is complicated, considering the efficacy, toxicity, and cost of available treatments. The authors conclude that second salvage autologous stem cell transplantation has a favorable risk/benefit profile in relapsed multiple myeloma and can be considered a viable treatment option for appropriate patients.

## Data Availability

The original contributions presented in the study are included in the article/supplementary material, further inquiries can be directed to the corresponding author.
